# Evaluation of Deployment Challenges of Wireless Sensor Networks at Signalized Intersections

**DOI:** 10.3390/s16071140

**Published:** 2016-07-22

**Authors:** Leyre Azpilicueta, Peio López-Iturri, Erik Aguirre, Carlos Martínez, José Javier Astrain, Jesús Villadangos, Francisco Falcone

**Affiliations:** 1 School of Engineering and Sciences, Tecnologico de Monterrey, Tecnologico 64849, Mexico; 2 Electrical and Electronic Engineering Department, Public University of Navarre, Pamplona 31006, Spain; peio.lopez@unavarra.es (P.L.-I.); aguirrerik@gmail.com (E.A.); martinez.61368@e.unavarra.es (C.M.); francisco.falcone@unavarra.es (F.F.); 3 Mathematical Engineering and Computer Science Department, Institute of Smart Cities, Public University of Navarre, Pamplona 31006, Spain; josej.astrain@unavarra.es (J.J.A.); jesusv@unavarra.es (J.V.)

**Keywords:** traffic lights, propagation, wireless sensor networks, ray launching

## Abstract

With the growing demand of Intelligent Transportation Systems (ITS) for safer and more efficient transportation, research on and development of such vehicular communication systems have increased considerably in the last years. The use of wireless networks in vehicular environments has grown exponentially. However, it is highly important to analyze radio propagation prior to the deployment of a wireless sensor network in such complex scenarios. In this work, the radio wave characterization for ISM 2.4 GHz and 5 GHz Wireless Sensor Networks (WSNs) deployed taking advantage of the existence of traffic light infrastructure has been assessed. By means of an in-house developed 3D ray launching algorithm, the impact of topology as well as urban morphology of the environment has been analyzed, emulating the realistic operation in the framework of the scenario. The complexity of the scenario, which is an intersection city area with traffic lights, vehicles, people, buildings, vegetation and urban environment, makes necessary the channel characterization with accurate models before the deployment of wireless networks. A measurement campaign has been conducted emulating the interaction of the system, in the vicinity of pedestrians as well as nearby vehicles. A real time interactive application has been developed and tested in order to visualize and monitor traffic as well as pedestrian user location and behavior. Results show that the use of deterministic tools in WSN deployment can aid in providing optimal layouts in terms of coverage, capacity and energy efficiency of the network.

## 1. Introduction

In the past two decades, the number of vehicles circulating in cities has increased considerably. This fact, together with the tendency of citizens to cluster has caused congestion of roads and junctions, especially in cities with large populations. The existing infrastructures have become inefficient to optimize traffic. The traditional traffic control system based on traffic lights depending on preset date and time patterns is not efficient in reducing waiting times and energy consumption. In fact, simulations show that adaptive traffic control based on information from Wireless Sensor Networks (WSNs) can be improved by 65% in normal traffic conditions compared to traffic management by preset patterns [1]. Since the nineties, there has been extensive research looking for different solutions in Intelligent Transportation Systems (ITS), with the objective of improving and performing traffic management. ITS consist of vehicle-to-vehicle (V2V) and vehicle-to-infrastructure (V2I) over wireless communications links, contributing to safety improvements and environmentally friendly driving. The ITS road infrastructure has the potential to enhance road safety by helping drivers avoid collisions during basic maneuvers, changing lanes, merging on highways, and driving safely in blind turns. The first implemented ITS were based on counting vehicles through magnetic sensors or artificial vision systems. However, these systems were not completely efficient because line of sight conditions must be fulfilled. Magnetometers sensors based on the detection of magnetic fields on the road lanes have been used to detect the presence of vehicles. These sensors can detect the number of vehicles and their speed, and send their data to the nearest Intersection Control Agent (ICA) which, determines the flow model of the intersection depending on sensors’ data, using ZigBee technology [2,3]. For the network architecture, there have been several proposals, ranging from a two sensors architecture [4,5] to several sensors with an ad-hoc network [6,7].

In the past few years, due to the advancement of wireless communications systems, there are new possibilities to manage traffic based on vehicle communications, which make it easier and more accurate than traditional approaches of detecting and counting vehicles. By adding short-range wireless communication capabilities to vehicles, the vehicles form mobile ad-hoc networks. These are referred to in the literature as Vehicular Ad-Hoc Networks (VANETs). Traffic safety is the main focus of current research on VANETs and the main motivation of deploying this technology making it ubiquitous. However, there are a number of other applications that could improve the way we drive today. In the future, the main goal for vehicles is to gather sensor data and share information on traffic dynamics with each other and with the road infrastructure.

Traffic lights are regarded as one of the most effective ways to alleviate traffic congestion. However, traditional traffic lights cannot meet the challenges in traffic regulation posed by the fast growing number of vehicles and increasing complexity of road conditions. Because of that, there are many works in the literature that deal with this problem. The work presented in [8] designs an adaptive traffic light system based on short-range wireless communication between vehicles, showing clear benefits compared to adaptive systems based on sensors or cameras. In reference [9], an intelligent dynamic traffic light sequence using radio frequency identification (RFID), which avoids problems that usually arise with standard traffic control systems, especially those related to image processing and beam interruption techniques is described. In [10], a new real-time traffic monitoring is proposed, based on a cluster V2X traffic data collection mechanism, which is able to gather more than 99% of the available data and reduce the overhead to one quarter when compared to other approaches. An intelligent traffic control system to pass emergency vehicles smoothly based on RFID and ZigBee technologies is presented in [11]. In [12], a ZigBee-based wireless system is also presented to assist traffic flow on arterial urban roads. The work proposed in [13] describes a dynamic traffic regulation method based on virtual traffic light for VANETs. The results demonstrate the viability of their solution in reducing waiting time and improving the traffic efficiency.

The operation of WSN is clearly dependent upon having adequate signal levels at the distributed nodes. When designing any wireless network, a significant issue to be considered is the maximum distance between two nodes that still ensures a reliable wireless connection. This depends on a broad range of parameters such as the transmitter power, the receiver sensitivity, the signal propagation environment, the signal frequency and the antennas parameters. In addition, the main challenge for vehicular communications is the rapidly changing radio propagation conditions. Both the transmitter and the receiver can be mobile, and the scattering environment can rapidly change. Due to this fact, the proper design of a WSN for use in these type of environments could be a very challenging process [14]. There are several papers in the literature that present WSN placement optimization mechanisms. In [15] a mechanism for deploying a minimum number of sensors to cover all targets that are randomly placed in a grid environment is presented. Reference [16] proposed a node deployment strategy for blindness avoiding in WSNs on the basis of ant colony optimization. The paper [17] investigates the problem of minimizing the total cost of deploying roadside units and sensor nodes along the two sides and the median island of a two-lane road to cover the whole road, and to form a connected VANET-sensor network. Reference [18] provides analytical modeling for IEEE 802.15.4-compliant WSN in vehicular communications, as well as guide design decisions and tradeoffs for WSN-based VANET applications. In [19], a numerical functional extreme model is proposed for searching the minimum exposure path in WSNs with a hybrid genetic algorithm. Reference [20] also proposes an addressing-based routing optimization scheme for IPv6 over low-power wireless personal area network in vehicular scenarios.

Different communication channel modeling approaches can be roughly split into four groups. The first one includes the empirical methods, which were used traditionally for initial coverage estimation (such as COST-231, Walfish-Bertoni, Okumura-Hata, etc.). Their advantage is that they provide rapid results, but they require calibration based on measurements to give an adequate fit of the results based on initial regression methods. The second group are the stochastic channel models (narrowband or wideband) [21], which characterize the channel from a frequency selective perspective. The third group includes the geometry-based stochastic models, which are the most widely used for propagation prediction in mobile communication channels, e.g., the work presented in [22] presents a V2V geometrical channel model considering that the local scatters move with random velocities in random directions. In [23], a new geometric-stochastic channel modeling is presented in which the delay-dependent Doppler probability density functions (pdfs) are derived for general V2V propagation environments, to cope with the non-stationarity of these channels. In reference [24], Pätzold and Borhani presented a non-stationary multipath fading channel model incorporating the effect of velocity variations of the mobile station. Reference [25] presents an adaptive reduced-rank estimation of non-stationary time-variant channels using subspace selection. However, none of the approaches described above consider all the elements of the environment, and because of that, they could fail in specific situations when the surroundings have a great impact on the electromagnetic propagation, such as a complex intersection in a large city, which could have vegetation, different type of scatters including different types of traffic lights, buildings, walking citizens, different materials, etc. Because of that, the fourth group of channel modeling approaches corresponds to deterministic methods, which are widely used for propagation prediction given a specific environment. They can be roughly divided into two categories: those which are based on ray optics (ray launching (RL) or ray tracing (RT) techniques) or the full-wave simulation techniques based on solving the Maxwell’s equations (method of moment (MoM), finite difference time domain (FDTD), etc.). These methods are precise but are time-consuming due to inherent computational complexity. Thus, methods based on geometrical optics (ray launching or ray tracing) offer a reasonable trade-off between precision and required calculation time, for radio planning purposes, with strong diffractive elements [26]. The main difference between the RL and RT techniques is that in the RL technique, rays are launched from a transmitter and, at the locations where rays intersect and object; the new reflected, transmitted, diffracted, or scattered rays begin. On the other hand, in the RT approach, the number of possible paths that rays follow from the transmitter to the receiver are encountered by imaging techniques.

In this work, deterministic modeling, specifically a RL technique, has been used to characterize the physical channel for radio planning purposes in an urban area for a context-aware system that benefits from traffic light infrastructure, given the ease of deployment as well as the vicinity to users under analysis, i.e., pedestrians as well as vehicles. Such system can obtain information in relation with pedestrian behavior (e.g., density of users in pedestrian crossings, pedestrian speed, incidentals in pedestrian movement), vehicle behavior or ambient information, among others. One of the main goals is to model the radio wave propagation adequately in order to optimize the distance between devices in an actual network deployment, by means of an in-house generated application which has been tested under real conditions. A real time monitoring application has been implemented in order to provide context-aware interactivity within the scenario under analysis, in relation with pedestrian/vehicle movement as well as environmental information which can be correlated with pedestrian/vehicle behavior. This paper is divided into the following sections: Section 2 describes the simulation procedure for the channel characterization of the considered scenarios and the scenarios description. Section 3 shows simulation results such as bi-dimensional planes of received power, power delay profiles and delay spread, among others. Section 4 presents the experimental-setup in the real scenario and the comparison between simulation and measurements. Section 5 describes the radio planning analysis for different technologies (ZigBee, Bluetooth and the 802.11p radio standard). Section 6 describes the proposed application within the considered scenario, followed by the last section presenting concluding remarks.

## 2. Ray Launching Technique and Simulation Scenarios

### 2.1. Ray Launching Technique

**Figure 1:**
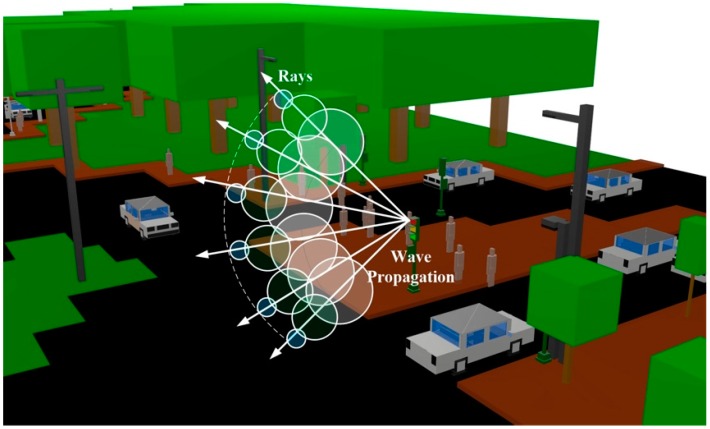
Wave front propagation with rays associated with single wave front points in the considered scenario.

A deterministic method based on an in-house developed 3-D RL code has been used to analyze the radio electric behavior of the considered scenario. The 3D RL algorithm is based on Geometrical Optics (GO) and Geometrical Theory of Diffraction (GTD). It has been verified in the literature with different applications, such as the analysis of wireless propagation in closed environments [27,28,29,30], interference analysis [31] or electromagnetic dosimetry evaluation in wireless systems [32]. The main principle of the RL techniques is to identify a single point on the wave front of the radiated wave with a ray that propagates in the space following a combination of optic and electromagnetic theories. This phenomenon is illustrated in Figure 1 for the considered scenario. Each ray propagates in the space as a single optic ray. The incident electric field (*E_i_*) created by an antenna at a distance r in the free space can be calculated by [33]:

(1) $$E_{i}^{⊥∥}=\sqrt{\frac{P_{\text{rad}}D_{t}\left(\theta_{t},\;\phi_{t}\right)\eta_{0}}{2\pi }}\frac{e^{-j\beta_{0}r}}{r}X^{⊥∥}L^{⊥∥}$$

where $P_{\text{rad}}$ is the radiated power with a directivity $D_{t}\left(\theta_{t},\phi_{t}\right)$, where the sub-index *t* refers to the transmitted angle, and polarization ratio $\left(X^{⊥},X^{∥}\right)$. $\beta_{0}=2\pi f_{c}\sqrt{\varepsilon_{0}\mu_{0}}$, $\varepsilon_{0}=8.854\times 10^{-12}$, $\mu_{0}=4\pi \times 10^{-7}$ and $\eta_{0}=120\pi .$ $L^{⊥∥}$ are the path loss coefficients for each polarization. The parameter *j* in Equation (1) refers to the complex number.

When the rays impinge with an obstacle in their path, a reflected and a transmitted ray are created with new angles provided by Snell’s law [34]. It is important to take into account that the rays considered in the GO only approach are only direct, reflected and refracted rays, leading to the existence of abrupt areas, which correspond to the boundaries of the regions where these rays exist. Because of that, the diffracted rays are introduced with the GTD and its uniform extension, the Uniform GTD (UTD). The main purpose of these diffracted rays is to remove field discontinuities and to introduce proper field corrections, especially in the zero-field regions predicted by GO. The diffracted field is calculated by [35]:

(2) $$E_{\text{UTD}}=e_{0}\frac{e^{-jks_{1}}}{s_{1}}D^{⊥∥}\sqrt{\frac{s_{1}}{s_{2}\left(s_{1}+s_{2}\right)}}e^{-jks_{2}}$$

where $s_{1}$, $s_{2}$ are the distances from the source to the edge and from the edge to the receiver point, respectively. $D^{⊥∥}$ are the diffraction coefficients given by [35,36].

**Figure 2:**
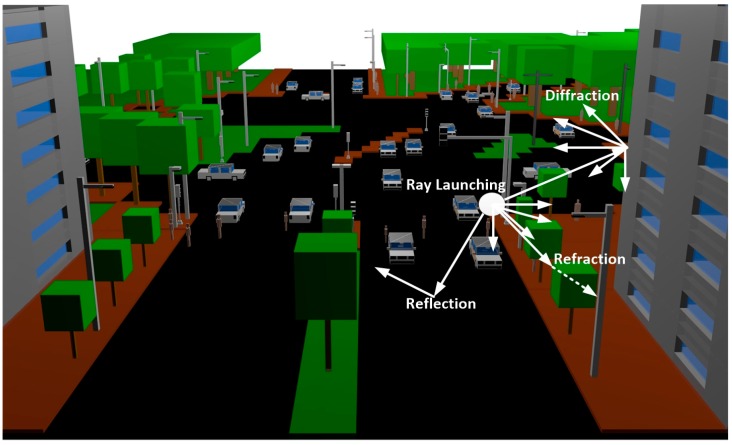
Schematic representation of the principle of operation of the in-house developed 3D RL algorithm.

The RL algorithm is performed three-dimensionally, with angular resolution (horizontal and vertical planes) in a predefined solid angle that considers the radiation diagram of the transceivers sources. Spatial resolution is also defined by a uniform hexahedral mesh. Parameters such as frequency of operation, radiation patterns of the antennas, number of multipath reflections, separation angle between rays, and cuboid dimension can be taken into account. Besides, all the material properties for all the elements within the scenario can also be considered, given the dielectric constant and the loss tangent at the frequency range of operation of the system under analysis. When a ray impacts with an obstacle, reflection, refraction and diffraction will occur, depending on the geometry and the electric properties of the object, as is depicted in Figure 2.

### 2.2. Simulation Scenario Description

**Figure 3:**
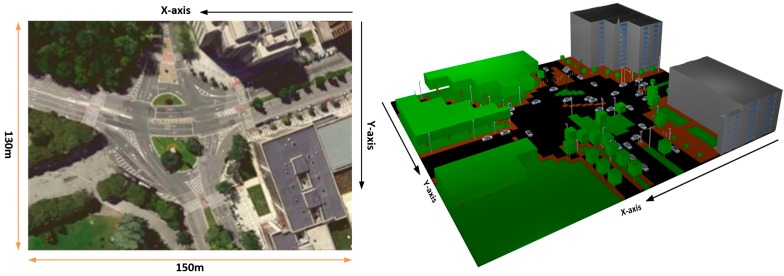
Real (**left**) and schematic (**right**) view of the considered scenario for simulation in the 3D Ray Launching Algorithm.

The simulation scenario implemented for calculation by means of the in-house developed 3D RL code consists of an outdoor environment of an urban city area, placed at the old town of Pamplona (Navarre, Spain). The considered scenario consists of an outdoor environment with trees of different heights, grass, junctions of roads, cars, people and different types of buildings. This environment can be considered as really complex, in terms of radio wave propagation, because it has a broad range of elements with different material properties within it. The dimensions of the scenario are (150 m, 130 m, 30 m). Figure 3 presents a view of the real and schematic considered scenarios.

All the material properties for all the elements within the scenarios have been considered, given the dielectric constant and the loss tangent at the frequency range of operation of the system under analysis. Taking into account that radio wave propagation in this scenario could change depending of the weather, as it is an outdoor environment, it is relevant to consider different conditions for the material properties of the vegetation. For that purpose, the values obtained in [37] for the material properties of the wood and the foliage of the vegetation have been used. A human body model [32], which take into account all the elements within the human body, and a car model [38], which also consider all the elements within a car, has also been used in the simulations. These models have been created to be implemented in the in-house 3D RL algorithm. The material parameters used in the simulation are defined in Table 1 [37,38,39,40].

## 3. Simulation Results

Once the simulation scenario has been defined, simulation results can be obtained. These results have been validated with real measurements which have been made in a campaign of measurements in the same scenario, which is shown in Section 4.

**Figure 4:**
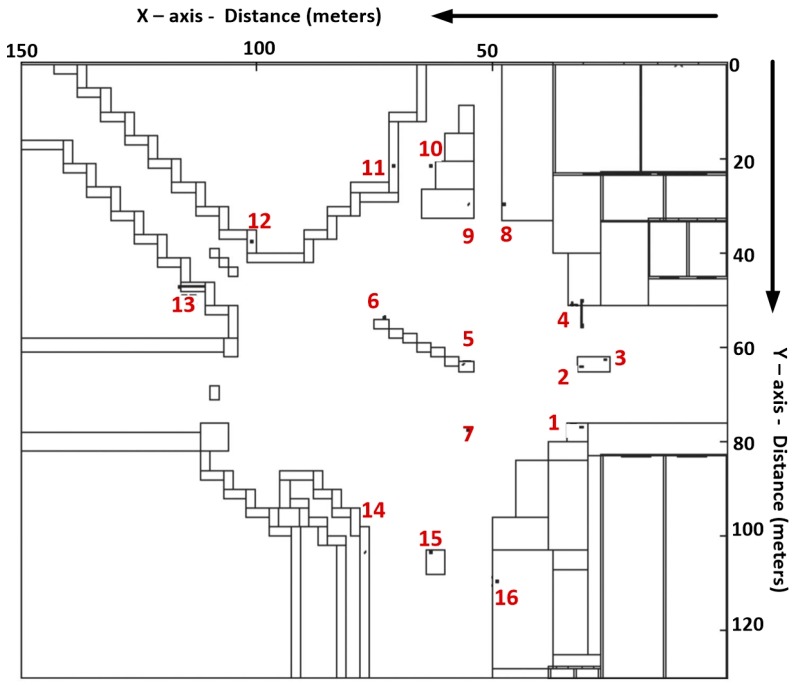
Schematic view of the position of the different antennas within the considered scenario.

For the simulations, 16 antennas have been placed in the considered scenario, one for each traffic light. The specific position of the antennas in the scenario is shown in Figure 4. To reduce interferences with people and vehicles, each antenna has been placed 10 cm above each traffic light. The radiating element is a wireless ZigBee mote which has been configured as a dipole, transmitting 18 dBm at 2.41 GHz. Simulation parameters are shown in Table 2.

**Figure 5:**
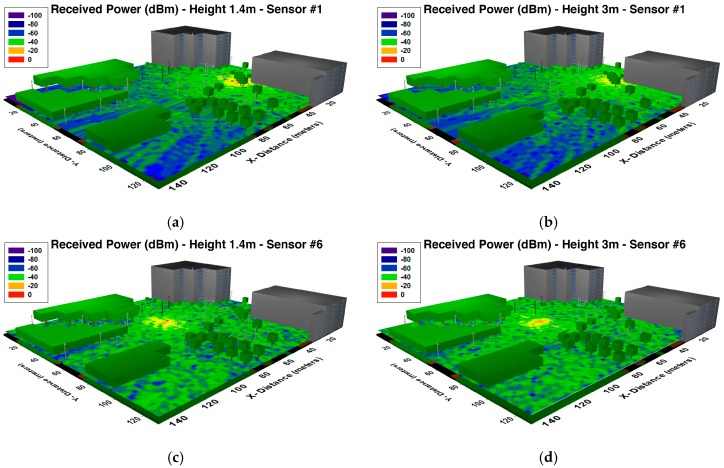
Estimation of received power (dBm) on the considered scenario (*XY* planes) for different heights obtained by the 3D Ray Launching algorithm (**a**) 1.4 m height for the sensor #1; (**b**) 3 m height for sensor #1; (**c**) 1.4 m height for sensor #6; (**d**) 3 m height for sensor #6.

**Figure 6:**
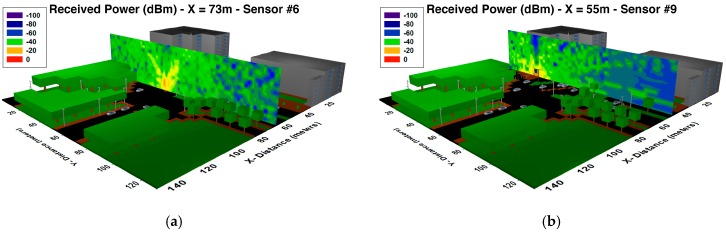
Estimation of received power (dBm) on the considered scenario (*YZ* planes) for different distances of *X* obtained by the 3D Ray Launching algorithm (**a**) *X* = 73 m for the sensor #6; (**b**) *X* = 55 m for sensor #9.

Figure 5 and Figure 6 shows the power distribution within the considered scenario for different heights and planes, and for different sensors placed in different traffic lights. As it can be seen, the influence of the obstacles (like the trees, streetlights, buildings, etc.) can be easily appreciated. It is shown that the morphology as well as the topology of the considered scenario has a noticeable impact on radio wave propagation.

**Figure 7:**
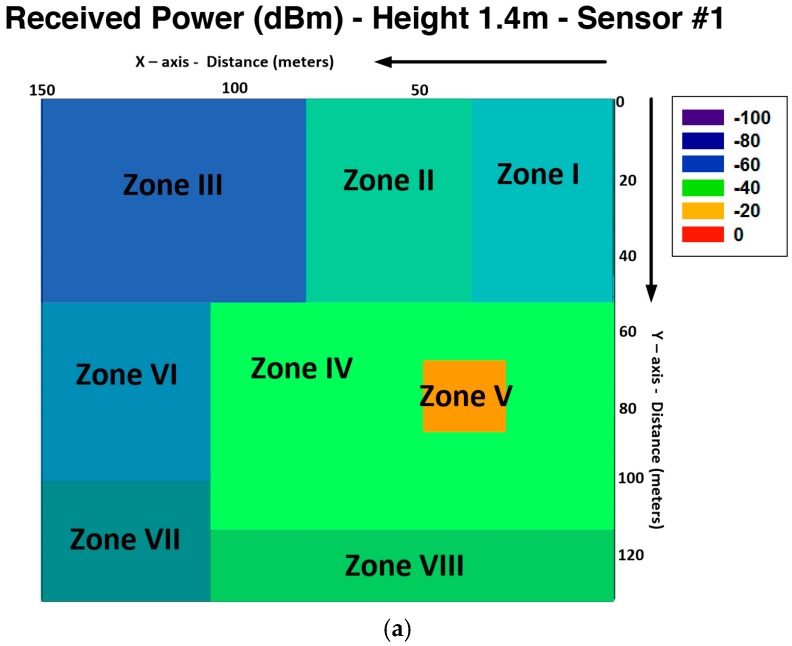

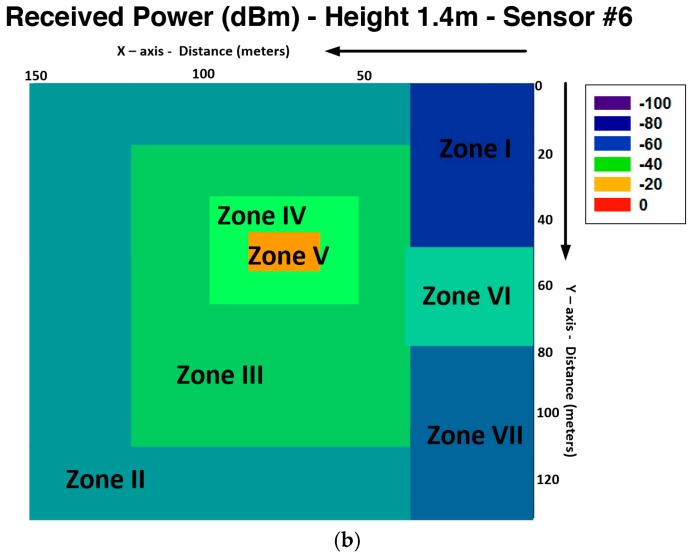
Estimation of received power (dBm) on the considered scenario (*XY* planes) divided by zones obtained by the 3D Ray Launching algorithm (**a**) 1.4 m height for the sensor #1; (**b**) 1.4 m height for sensor #6.

Figure 7 shows the different zones of estimated received power extracted from the data of Figure 5a,c. It can be seen that received power results are accurate but not homogeneous, and because of that, the radio planning task has an important role. Different values of the mean received power are obtained depending of the obstacle density of the different zones. Table 3 show the different values of power mean with its standard deviation and the obstacle density of the zone considered. It can be seen that the obstacle density has great influence in received power, but it is also relevant to consider the area of the considered zone and the distance from the transmitter of each zone. From these results, it is shown that received power in different points of the scenarios depends greatly of the position of the transmitter, the obstacle density of the different zones and the different material properties of the obstacles.

**Figure 8:**
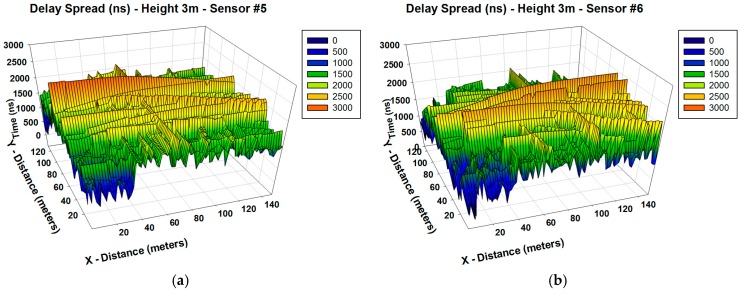
Estimation of delay spread (ns) on the considered scenario for different positions of the transmitter antenna in different traffic street lights (**a**) 3 m height for sensor #5; (**b**) 3 m height for sensor #6.

**Figure 9:**
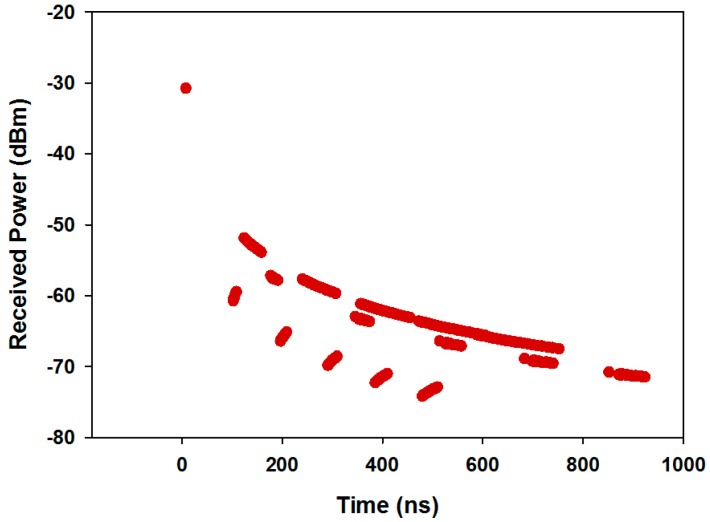
Power Delay Profile at a given cuboid, located at the central location in the considered scenario.

An important radioelectric phenomenon in this type of environment is given by multipath propagation. To illustrate this fact, the delay spread for the whole scenario and the power delay profile for the central location of the scenario has been obtained and it is shown in Figure 8 and Figure 9, respectively. The delay spread has been defined as the time delay between the first and the last ray which arrives to each spatial point. It has been calculated using as threshold the noise floor, with a value of −100 dBm.

From Figure 8 it can be seen that multipath phenomena have a lot of influence in the environment. It is observed that the delay spread is higher in the areas closest to the transmitter antenna and it is lower in other areas because the radiation power in these parts is smaller due to the larger distance. Each spatial sample of the delay spread is associated with a power delay profile, shown in Figure 9 for the central position of the scenario. As it is observed, the scenario is really complex and there are several echoes in the scenario due to this multipath channel behavior.

As previously shown, a clear dependence on the topology and morphology of the scenario as well as on the system parameters is observed and must be carefully considered in order to assess the performance of multiple systems within a particular scenario. These results allow the optimization of the design of the WSN since the designer can select the minimum number of nodes required to grant a certain communication level, and the optimal emplacements of these nodes. It is important to consider also that a larger density of nodes can lead to increased interference levels, which could degrade system performance. This comment also applies in the case of changes in the transmitted service, associated with a certain allocated bandwidth and hence, changes in sensitivity levels.

The computational time is also highly important when analyzing these complex scenarios. In this case, simulations have been performed in an Intel Xeon CPU X5650 @ 6.67 GHz and 2.66 GHz, and the simulation computational time has been of 43,537 s using the software Matlab from Mathworks (Natick, MA, USA). It must be pointed out that the input parameters have been chosen according to a convergence analysis of the algorithm previously done for different types of scenarios [41].

**Figure 10:**
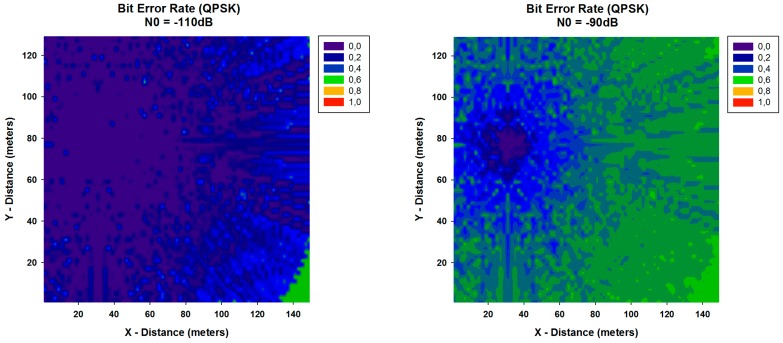
Bit Error Rate for QPSK modulation for different values of $N_{0}$ for data rate of 250 Kbps.

**Figure 11:**
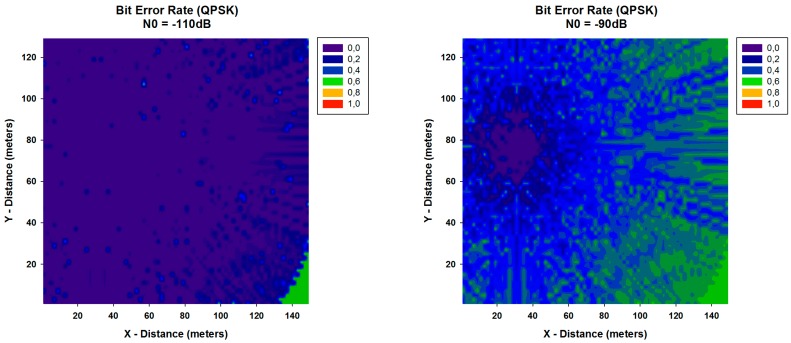
Bit Error Rate for QPSK modulation for different values of $N_{0}$ for data rate of 57,600 bps.

A key parameter that is used in assessing systems that transmit digital data from one location to another is the Bit Error Rate (BER). The *BER* expression for QPSK modulation can be calculated by:

(3) $$BER_{\text{QPSK}}=Q\left(\sqrt{2E_{b}/N_{0}}\right)$$

where $E_{b}=P_{\text{RX}}/R_{b}$ and *N*_0_ is the noise value. The received power $P_{\text{RX}}$ has been calculated with the 3D RL algorithm for each spatial sample in the considered scenario. With these values of $P_{\text{RX}}$, the *BER* has been calculated and it is shown in Figure 10 and Figure 11, for different values of data rate ($R_{b}$) and different values of $N_{0}.$ It can be seen the high variability between the different cases, with higher values of *BER* when $N_{0}$ is higher. In addition, differences between the different data rates considered are observed, leading to a lower BER for all cases with the lowest data rate considered. These results can be really helpful in order to optimize the design and deployment of a WSN depending on the modulation, the used data rate and the level of $N_{0}.$

## 4. Experimental Setup

**Figure 12:**
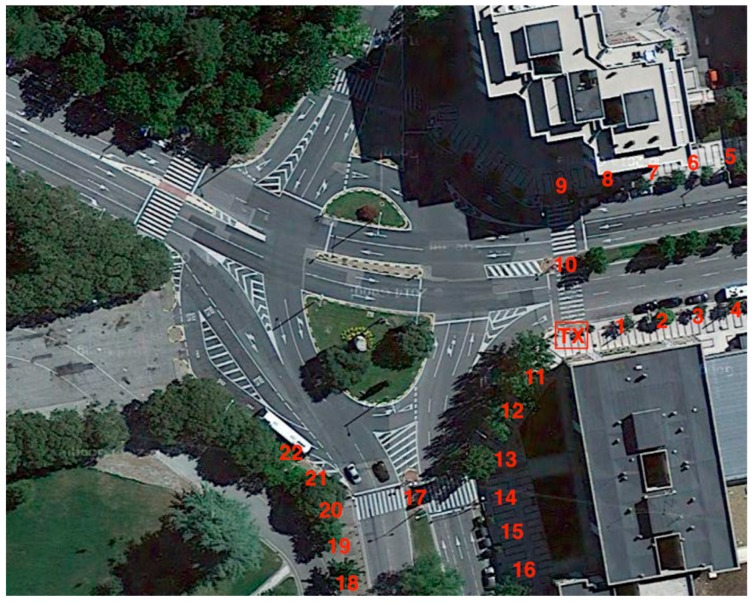
View of the considered scenario with the transmitter position and the measurement points.

In order to validate the results previously obtained, real measurements in the same scenario shown in Figure 3 have been performed. A transmitter antenna, connected to a signal generator at 2.41 GHz and 5.9 GHz has been located at the coordinates (*X* = 31 m, *Y* = 76.8 m, *Z* = 2.05 m), just above the traffic light #1. The employed signal generator is a portable N1996A (Agilent Technologies, Santa Clara, CA, USA) unit and the spectrum analyzer is an Agilent N9912 Field Fox. The antennas used are ACA-4HSRPP-2458 from Zentri (Los Gatos, CA, USA), both omnidirectional antennas, with a gain of 0.3 dB for 2.41 GHz and a gain of 3.74 dB for 5.9 GHz. Measurements have been performed along the measurement points depicted in Figure 12 each 5 m at a height of 1.4 m.

**Figure 13:**
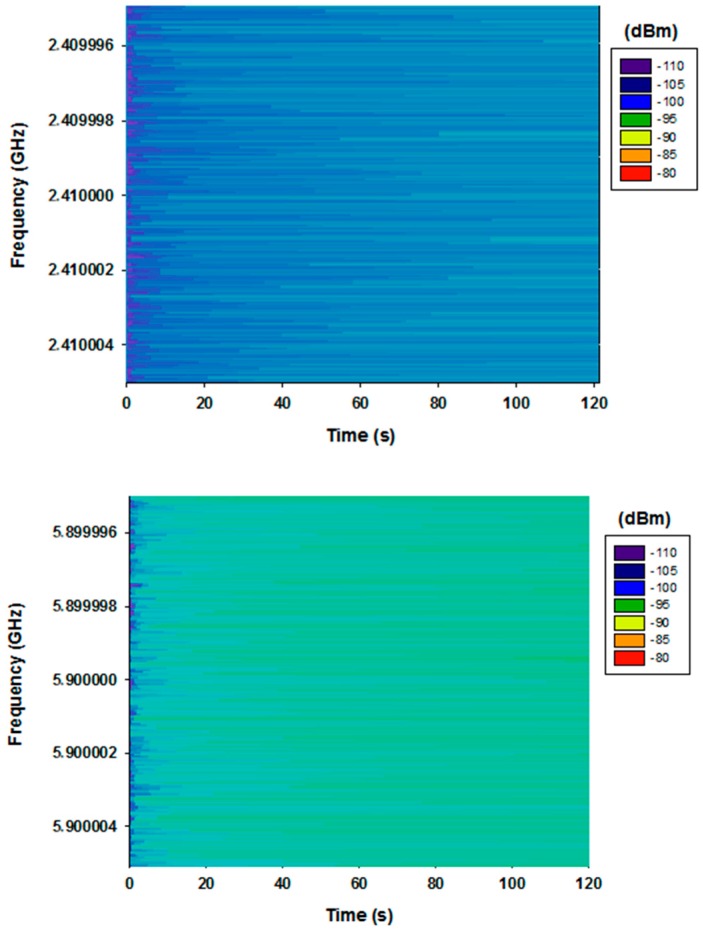
Measured spectrogram in the 2.41 GHz (**top**) and 5.9 GHz band (**bottom**).

To visualize the frequency variation with time and the possible influence of pre-existent interference in the scenario, a spectrogram has been measured before starting the measurement campaign. Figure 13 shows the measured spectrogram for both frequencies in Max Hold mode, for the transmitter position represented in Figure 12 in the considered scenario, with the aid of the Agilent N9912 Field Fox portable spectrum analyzer. 

It can be seen that the measured values can be considered as noise and there is not a significant pre-existent interference at the frequency bands of interest. It is also observed that the noise is slightly higher for 5.9 GHz frequency. This is due to the fact the gain of the antenna in 5.9 GHz is 3.44 dB higher than at 2.41 GHz frequency.

**Figure 14:**
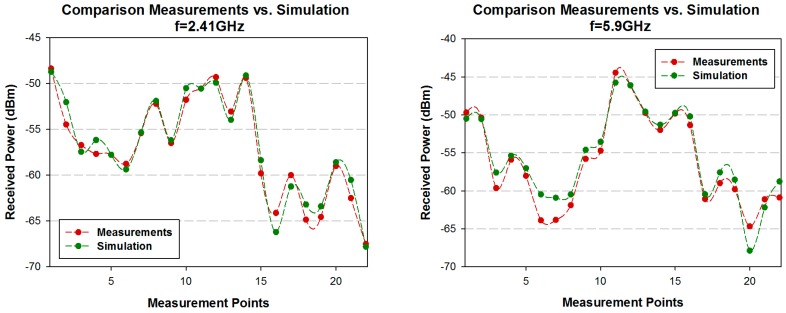
Comparison simulation vs. measurements for 2.41 GHz and 5.9 GHz in the scenario considered.

Figure 14 shows the comparison between simulation and measurement results for both frequencies. Measurements were performed with 100 MHz bandwidth at 2.41 GHz and 5.9 GHz frequency. The measurement time at each point was 60 s, and the value of received power represented by each point is the highest peak of power shown by the spectrum analyzer for the considered bandwidth (MaxHold function in the spectrum analyzer of Agilent). The received power values estimated by simulation have been obtained for the same spatial samples as the real measurements, considering the corresponding cuboid in the three-dimensional mesh of cuboids in which the scenario have been divided. It is observed a good agreement between simulation and measurement results, with a mean error of 0.27 dBm with a standard deviation of 1.13 dB for 2.41 GHz frequency, and 0.66 dBm with a standard deviation of 1.46 dB for 5.9 GHz frequency. The resulting error means are very low, indicating that the proposed 3D RL simulation method works properly, validating in the same way the simulation results shown in the previous sections of this work.

## 5. Radioplanning Analysis

The deployment of three different communication systems has been analyzed within the considered scenario. Firstly, an analysis of the received power and the receiver’s sensitivity for the different technologies has been analyzed, leading to the most efficient coverage ratio for the deployment of the different systems. The first technology evaluated has been ZigBee technology. Specifically, the wireless devices used for simulation have been both, the XBee Pro and XBee motes from Digi International Inc. (Minnetonka, MN, USA). The main difference between them is the larger transmitter power of the XBee Pro, leading to longer range distance. In order to analyze the worst case conditions, the transmitted power levels considered has been reduced to the minimum default value. After that, the second technology evaluated has been Bluetooth, specifically Bluetooth Low Energy (BLE) and classic Bluetooth. BLE has been designed as a low-power solution for control and monitoring applications, in contrast with classic Bluetooth. Finally, the third technology evaluated has been the 802.11p radio, which is the standard to add wireless access in vehicular environments (WAVE). It must be pointed out that for this technology we have performed new simulations in the 5.9 GHz frequency. Three different modulations with three different data rates have been analyzed for the 802.11p radio standard. The transmitted power and sensitivity for the different communication systems considered is shown in Table 4.

**Figure 15:**
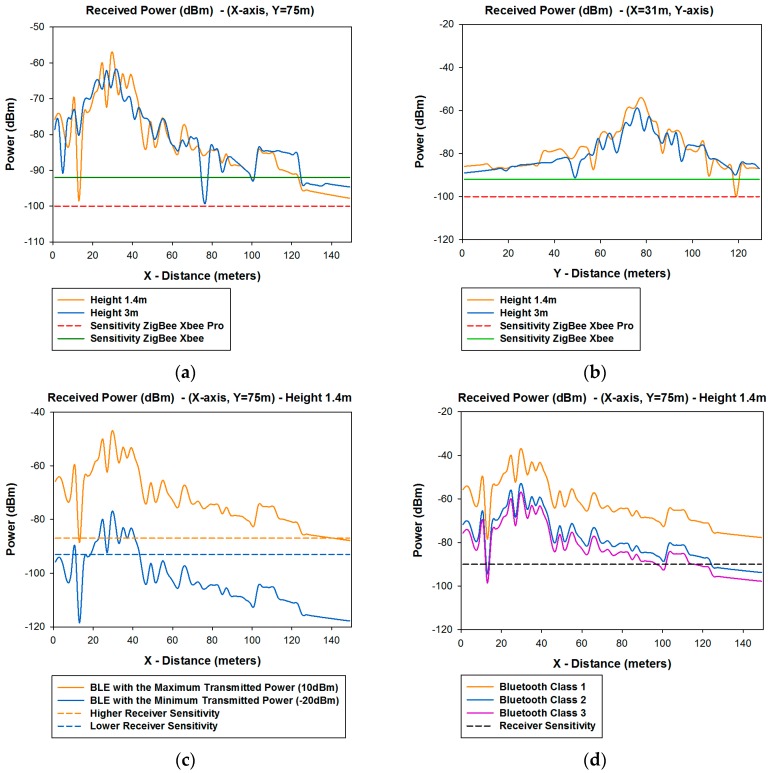

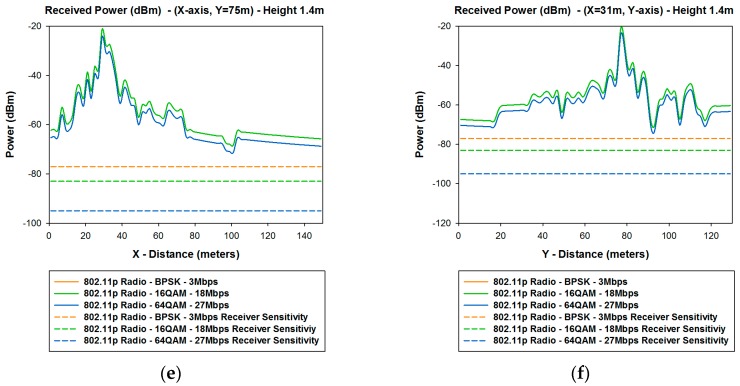
Comparison of radial of received power (dBm) along the *X*-axis with the receiver sensitivity (**a**) ZigBee XBee Pro and ZigBee XBee for *Y* = 6 m; (**b**) ZigBee XbEe Pro and ZigBee XBee for *Y* = 14 m; (**c**) BLE system; (**d**) Classic Bluetooth; (**e**) 802.11p Radio for *Y* = 6 m; (**f**) 802.11p Radio for *Y* = 14 m.

**Figure 16:**
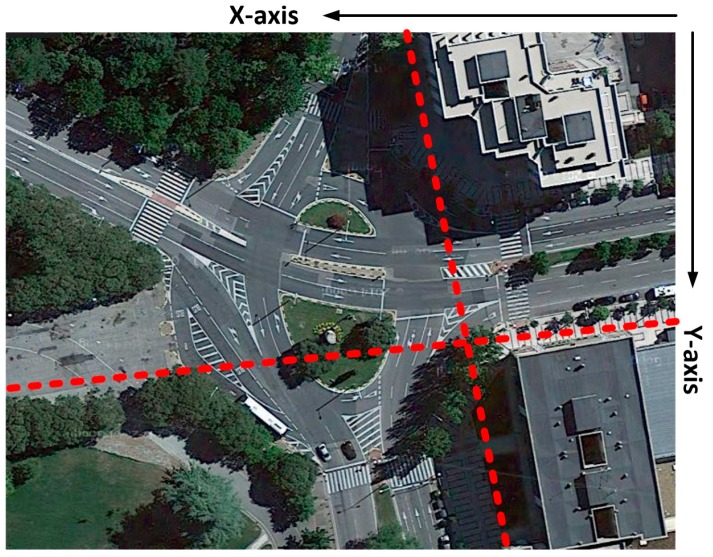
Aerial view of the radial lines, which are represented in Figure 15, along the *X*-axis and *Y*-axis.

Figure 15 shows the linear distributions of received power along the *X*-axis for different values of *Y* for different heights in the case of ZigBee and 802.11p radio and for 1.4 m height in the case of Bluetooth. In order to clarify which distribution lines are those represented in Figure 15 in the real scenario, the aerial view of these distribution lines are shown in Figure 16. It is observed that the distribution lines of received power, which corresponds with the transmitter antenna placed at the traffic light #1 are represented. These simulations results are shown in a bi-dimensional plane in Figure 5a. 

Figure 15a,b shows the comparison between the received power of XBee Pro and XBee with the sensitivity of each of them. Figure 15c shows the comparison between BLE with the maximum and minimum transmitted power and the higher and lower receiver sensitivity depending of which type of BLE has being used. Figure 15d shows the comparison between Class 1, Class 2, and Class 3 of classic Bluetooth. Figure 15e,f shows the comparison between different devices with different modulations and data rates of the 802.11p standard, for different values of *Y*. It can be seen that there are some points where the signal goes down below the sensitivity level.

**Figure 17:**
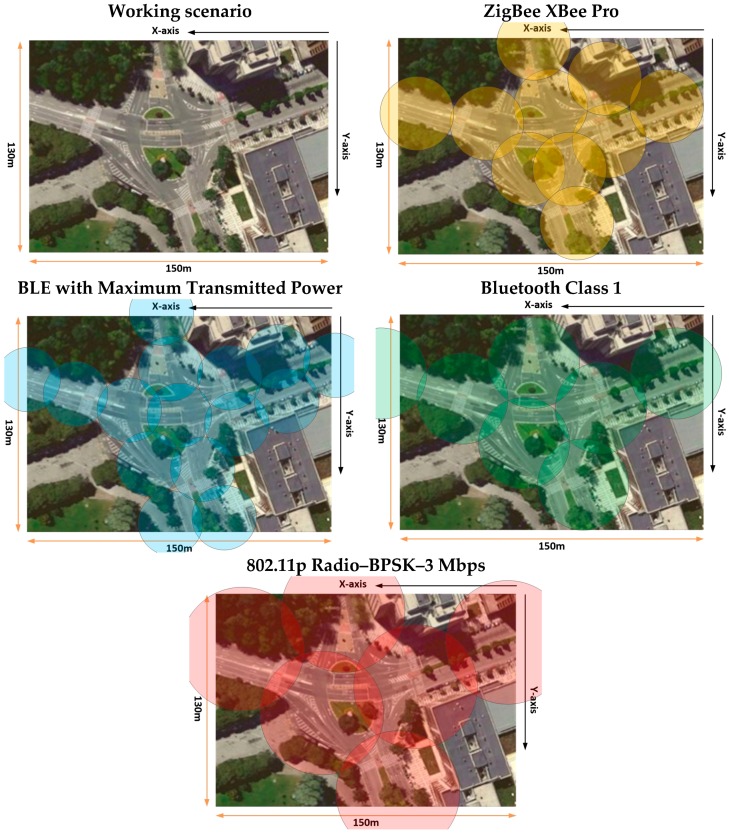
Radioplanning coverage for different technologies within the considered environment.

The comparison of the linear distribution of received power along the *X*-axis and *Y*-axis for different lines in the considered scenario with the different sensitivity levels, leads to radio planning engineers to know the radio coverage of the different systems. These results, along with the different planes of received power achieved for the whole scenario with the 3D Ray Launching Code, gives precise results but no homogeneous. As stated before and it is represented in Figure 7, the received power can be approximated for regular coverage zones. Depending of these results, and considering also the capacity of the system, an optimal design of the wireless sensor network coverage can be obtained. In order to represent a practical example, a schematic view of a final implementation with omnidirectional antennas for the different systems analyzed in the considered scenario is shown in Figure 17. It must be pointed out that the different radio coverage of the different circles has been obtained according to simulation results represented in Figure 15, and also taking into account the complete planes of received power, which have been obtained for the whole scenario and several examples have been represented in Figure 5 (*XY* planes), Figure 6 (*YZ* planes) and Figure 7 (*XY* planes divided by zones).

**Figure 18:**
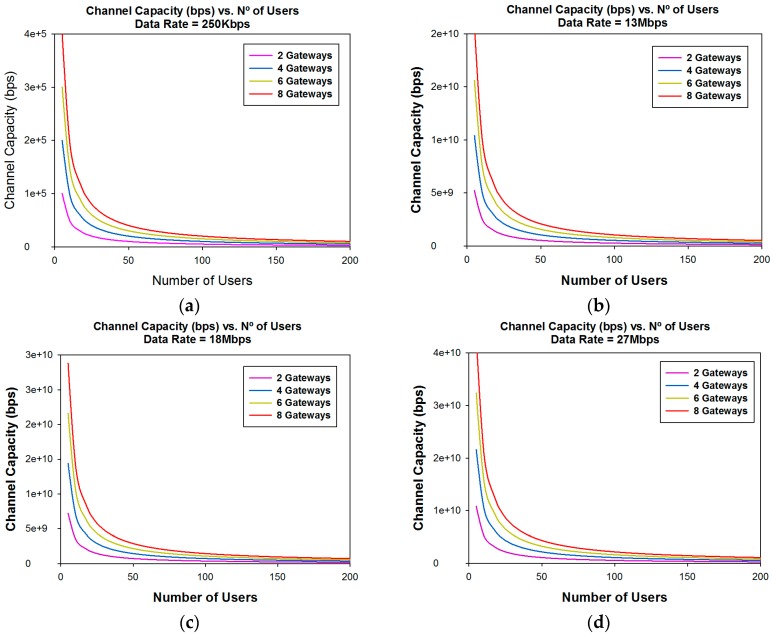
Channel capacity (bps) vs. the number of users for different number of gateways considered. (**a**) Data Rate = 250 Kbps; (**b**) Data Rate = 13 Mbps; (**c**) Data Rate = 18 Mbps; (**d**) Data Rate = 27 Mbps.

**Figure 19:**
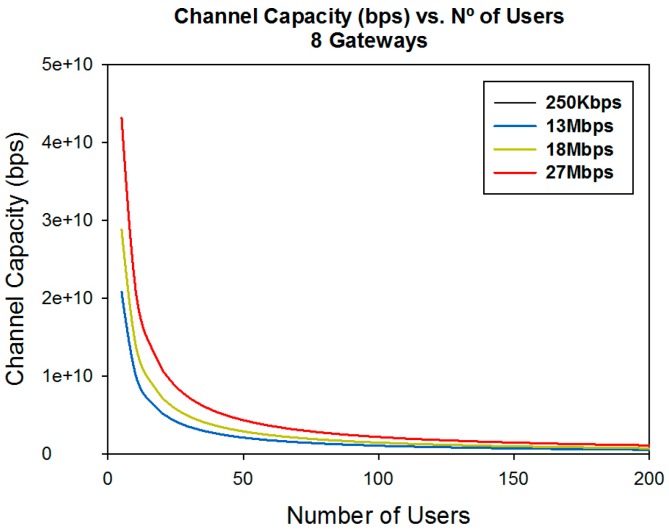
Channel capacity (bps) vs. the number of users for different data rates for eight gateways considered.

Another important parameter which is important when designing a WSN is capacity. Once coverage levels are satisfied, we must have adequate capacity in order to have a good quality of service in the communication link. The channel capacity depends on the number of users who are connected to the same communication link at the same time, the data rate of the transceivers and the number of gateways in which the information is gathered. Figure 18 and Figure 19 show the channel capacity vs. the number of users for different data rates considered and for different number of gateways. It is observed as expected that the channel capacity increases for the same number of users, for every data rate considered, if the information is gathered with a higher number of gateways.

The great difference in the channel capacity depending on the data rate considered is also very significant. This leads us to conclude that it is highly important to adequately fix the number of gateways in the design phase, depending of the data rate of the transceivers and the expected number of users.

## 6. Application

Once we have modeled the scenario in terms of potential restrictions given by wireless channel operation and the required radio-planning phase, the main goal of the system is to produce a visualization platform presenting a city’s traffic status on real time. The main stakeholder is the municipality. It is worth noting that the results previously obtained in Section 3 provide insight into the characteristics of wireless propagation for the scenario under analysis. In this way, estimation of path loss is derived, a required information in order to perform the radio-planning analysis which is described in Section 5. These results provide the density of nodes as a function of the employed transceiver. This information in turn is used in order to adequately dimension the applications which can be executed, as well as the location of the nodes within the context of the specific items of the application, such as pedestrian monitoring. The proposed application that has been implemented, its characteristics and the functional/system architecture will be described in this section.

The traffic in a city, as many other parameters, is very time/day dependent. Mobility patterns highly depend on the day and time of the day. Therefore, traffic light policy is a very complex task and difficult to execute in real time. Additionally, each decision at one place in the city impacts directly or indirectly on other areas of the city. It is a very complex task to maintain a fluid traffic flow in the whole city. In fact, it could be impossible in some situations like blocked streets due to meteorological issues.

In order to analyze the traffic in a city, usually the city is logically divided in areas. Each area defines a conflict region where cars, pedestrian and bicycles are inputs, which should be processed to get out of the conflict region (some of them could remain inside). The conflict area is modeled using queuing theory. Each cross is a join of streets at a common point. Then, each street is modeled as a queue and server, with a processing policy. Mainly, this policy is first-in-first-out (FIFO). Traffic lights are the components of the system that regulate the order to process inputs on queues (server order). In this model, the arrival process of the inputs is a key factor to analyze each conflict area. Such a model is applied mainly for worst and best case modeling. This approach models different conflict areas independently, and not the whole city.

In order to make decisions about traffic light policies, it is important to monitor the traffic, and to store historic data. Such data will be used to adjust the model: determine the arrival process, and the server processing time. However, such simulations are particular cases and while useful to analyze worst case, and average case, it is difficult to optimize general parameters like average time to cross the city.

In our case, we propose another option to analyze a city’s traffic by gathering traffic information on-line and presenting it on a visual interface. In this case, traffic managers could analyze in real time the situation of the traffic in the city. The collected information could be used to adjust the previous model (queue network model to analyze the traffic by conflict areas).

In our case, we gather such information based on a WSN. Each sensor is located at a traffic light. Each one collects information about the number of cars and the time interval between each pair of cars. Periodically, we integrate this information in a single packet, and transfer it to a central control, where data is stored and processed. In the initial test, we have deployed 20 nodes within the scenario under analysis, although there was no limitation to increase the network size, scalable as a function of the system requirements. In relation with the sensors deployed, we have focused mainly on weather, pollution (CO_2_ and NO) and presence. The employed nodes allow monitoring other items, such as multiple polluting substances, ambient conditions, liquid flow or existence of electric currents, to name a few.

The graphical interface has been implemented in order to illustrate the city’s traffic state with the aid of lines and different colors. We have parameterized our sensors to evaluate levels of congestion. We use two parameters to establish line’s color, and type (points or partial lines): arrival rate (AR), to count the number of cars arriving at the traffic light each time unit; and processing rate (PR), to measure the time the car requires to pass next to the traffic light.

**Figure 20:**
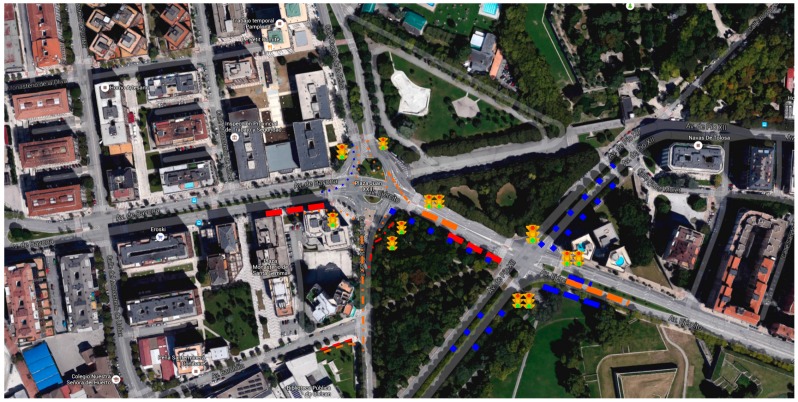
Traffic monitoring tool.

Both parameters are measured at the traffic light, and integrated periodically to evaluate the average value of the above parameters. Based on them, we evaluate the Congestion Threshold (CTh, as the ratio between AR and PR), which represents the level of congestion of a traffic lane. With blue lines we identify fluid traffic (CTh = 1, orange is used to denote partial congestion (1 < CTh < 5), and red means dense traffic (5 < CTh). The style of the line (points or small lines) denotes the arrival rate, using points to identify the case of low arrival rate. Figure 20 illustrates the traffic congestion at two different locations at the city of Pamplona.

### 6.1. Application Architecture

In our scenario we have deployed numerous sensors. Each one has common software to execute. Each node communicates its information to a common server. The server collects the information, and present the information to the users. As we have similar tasks at different locations, their results are collected in a common point, and it is not required any temporal restriction. We have deployed a message bus software architecture. The main advantage is the scalability of the approach, and minimization of application coupling. Each sensor can work independently.

**Figure 21:**
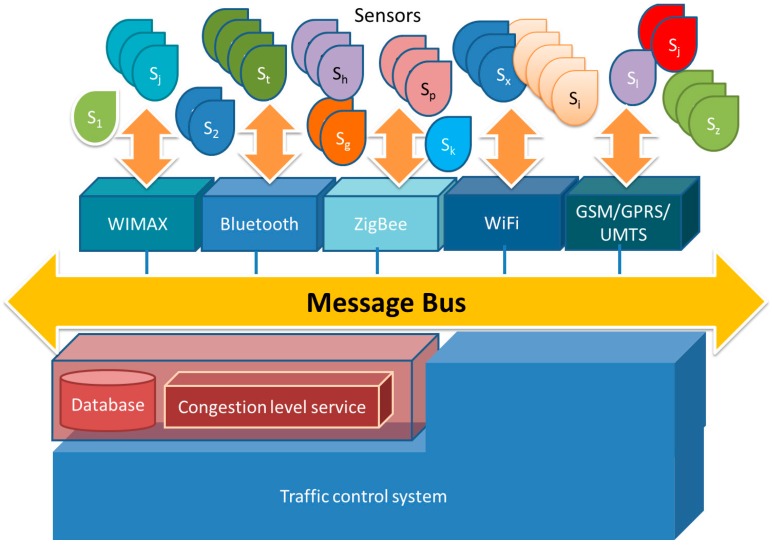
Message bus software architecture.

Figure 21 depicts the message bus architecture, which uses a software system that can receive and send messages using one or more communication channels, so that applications can interact without needing to know specific details about each other. Interaction between applications is accomplished by passing messages (asynchronously) over a common bus. We implement the message bus architecture using a messaging router, and it is implemented using message queuing.

The motes placed around the scenario collect traffic information from sensors (microwave radar, image processing, infrared and ultrasonic in our case) and introduce it into the system by means of the message bus. The information sensed is persistently stored into a database. Data collected is analyzed in order to detect traffic congestion levels. Such congestion levels are presented to experts (traffic managers) in order to allow them to take decisions within the existing traffic control systems. The congestion level service may be used to provide real-time information to experts or either it could be embedded as part of a traffic control system. Figure 20 shows the traffic monitoring tool that allows experts to visualize the traffic incidences observed by the sensor network. Making use of the publish/subscribe paradigm, the congestion level service pushes the traffic flows to those monitoring tools that have subscribed the service. In the same way, the traffic control system may also subscribe this service, so that the traffic control system has a new source of information to be considered in decision algorithms and internal processes.

**Figure 22:**
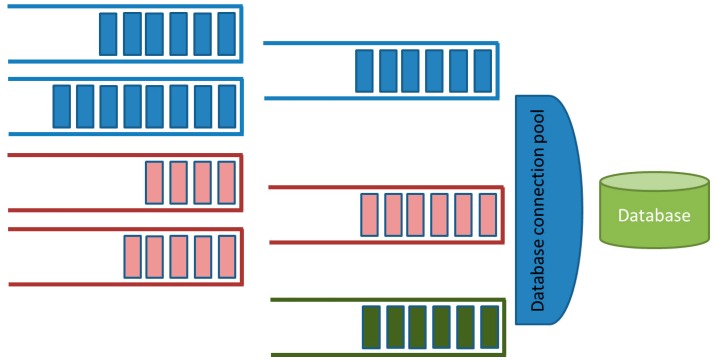
Queueing system and data insertion into the database.

Data integration is performed at motes. Each mote collects the information from sensors and aggregates this information in order to minimize the number and length of traffic messages. Each mote communicates the information, using a ZigBee interface, to a gateway. The gateway inserts the information on the message bus where a set of parallel process manage the existing queues and store the information into a database. The message bus includes a set of message queues geographically distributed in order to collect information according to the geographical location of the sensors. The queue hierarchy allows the arranged gathering of the traffic information, avoiding duplicated or incomplete information. A pool of database connections allows maximizing the data insertion rate into the database. Figure 22 depicts the gathering process.

The message bus uses services for communication between the bus and components (sensors) attached to the bus. In addition, the bus provides services that transform message from one format to another, allowing sensors using incompatible message formats to communicate with each other. In Figure 19 we can see the capacity of the system in terms of number of users. This means applications can be hosted in the cloud instead of on an enterprise network. The cloud improves the scalability of the system, because multiple instances of motes can be attached to the bus in order to handle multiple requests at the same time.

### 6.2. Congestion Level Service

The analysis of the information stored at the database is performed using a fuzzy algorithm developed with the aid of Matlab. We have trained an Adaptive Neuro-fuzzy Inference System (ANFIS) according to data collected from sensors and stored into the database.

The fuzzy system has two input and one output variables. The input variables are the traffic density observed during a given period (traversingVehiclesRate, number of vehicles observed from a traffic light during the green period); and the number of vehicles awaiting the next traffic light cycle (#waitingGreen). The congestion level is the output variable. The output values vary on the range [1,10] as described above. The membership degree of each variable is given by three fuzzy sets (low, medium and high) provided by the ANFIS. Table 5 briefly summarizes the decision rules.

In this paper, in order to illustrate congestion level service operation we have deployed a simplified interface, where data collected is used to present the current status of the traffic in the city. However, such information is processed to augment the information at each point taking into account previous analysis.

**Figure 23:**
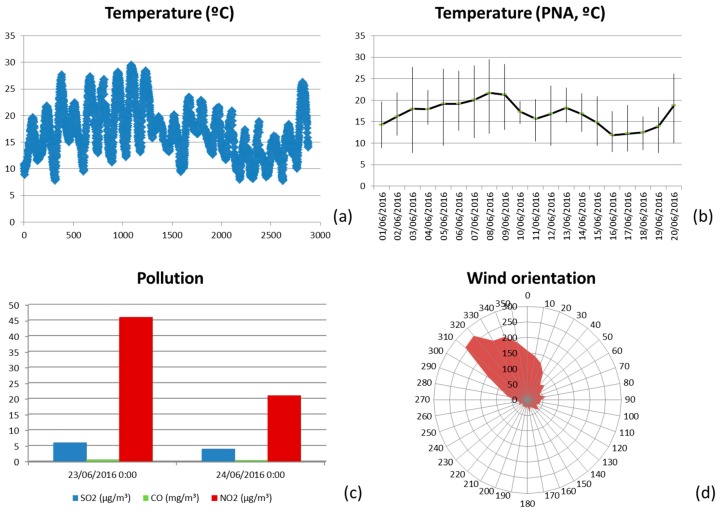
Parameters monitored by the WSN. (**a**) Temperature variation per hour; (**b**) Maximum, minimum and average temperatures per day; (**c**) Relative humidity per day; (**d**) Wind orientation and intensity.

**Figure 24:**
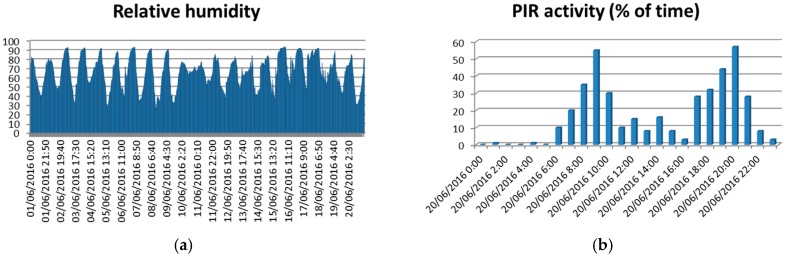
Relative humidity measured (**a**) and PIR activity detected (**b**).

In our case, a first approach has been the evaluation of the status of each conflict area considering the congestion level at different days. We integrate on a graphical view the level of congestion during different periods of time. We group the week on labor days (LD) and holidays (HD). We average the congestion level for both groups, and provide it to the user (traffic manager). In Figure 20 it corresponds to the pie chart next to some traffic lights. The colors of the pie chart correspond to the congestion level at each location. Figure 23 shows some of the parameters and indicators considered (temperature, wind orientation and intensity, pollution degree…). The average, maximum and minimum temperatures observed during the three first weeks of June 2016 are depicted in Figure 23b, while Figure 23a shows the variation of the temperature each hour during the same period. The graphs on the bottom show the pollution degree (CO, SO_2_ and NO_2_) and the wind direction (bottom right). Traffic managers can use such information in order to temporarily modify traffic light policies, or to send human resources (police) to regulate the traffic. Currently we are evaluating a few parameters, but the sensors deployed at traffic light have an enormous potential to provide better services for citizens. Nowadays, we obtain situational awareness from temperature and pollution sensors and also from passive infrared sensor (PIR), which aid to detect the presence of pedestrians and vehicles, allowing the estimation of the degree of occupancy of both lanes and crosswalks. Figure 24a shows the relative humidity observed, while the graph on the right shows the percentage of operating time during which the infrared sensor detects pedestrians. The system here proposed provides complete and adequate situational awareness to municipal experts in order to allow the modification of the traffic light cycles according to the specific needs of each instant considering multiple environmental factors in addition to the traditional vehicle counting.

## 7. Conclusions

In this work, the demands for modeling the radio channel in an urban city area for intelligent traffic light control systems are presented. The obtained results show the complexity of this type of scenario, with traffic lights, vehicles, people, buildings, vegetation and urban environment. It can be concluded that the morphology as well as the topology of the scenario play a key role in the estimation of radio signal propagation, due to the strong impact of multipath components in the overall loss mechanism of the propagating radio waves. The validation of the 3D Ray Launching algorithm has been done with a good match in the comparison with real measurements. The use of simulation tools, such as the 3D Ray Launching code, is a great aid for radioplanning analysis with the aim of deploying WSN within this type of environments. Moreover, the context aware scenario implementation using different wireless technologies can be properly designed with a detailed characterization and analysis of the radio wave propagation for different frequencies. The system has been tested by means of an ad hoc application and its corresponding platform, providing real time traffic monitoring within the scenario under analysis. Results show that the use of deterministic tools in WSN deployment can aid in providing optimal layouts in terms of coverage, capacity and energy efficiency of the network.

## Figures and Tables

**Table 1:** Material properties in the RL simulation.

Parameter	Permittivity (*ε_r_*)	Conductivity (*σ*) (S/m)
Air	1	0
Concrete	25	0.02
Aluminum	4.5	4 × 10^7^
Polypropylene	3	0.11
Brick	4.44	0.11
Glass	6.06	10^−12^
Plasterboard	2.02	0
Grass	30	0.01
Trunk Tree	[37]	[37]
Tree foliage	[37]	[37]
Human body model	[32]	[32]
Car model	[38]	[38]

**Table 2:** 3D Ray Launching simulation parameters.

Parameter	Value
Frequency	2.41 GHz
Transmitted Power Level	18 dBm
Vertical plane angle resolution ∆θ	2°
Horizontal plane angle resolution ∆φ	2°
Reflections	6
Cuboids size	2 m

**Table 3:** Simulation results divided by zones.

Zone	Area (m^2^)	Power Mean (dBm)	Standard Deviation (dB)	Obstacles Density (%)
**CASE I Sensor #1**				
**I**	700	−54.57	1.96	0.43
**II**	875	−53.27	1.70	0.57
**III**	3675	−68.47	9.09	0.04
**IV**	5600	−47.86	2.27	0.86
**V**	400	−36.81	5.16	0.10
**VI**	4550	−57.28	1.08	0.02
**VII**	2100	−58.96	2.22	0
**VIII**	1600	−52.27	1.14	0.26
**CASE II Sensor #6**				
**I**	1400	−65.10	5.61	0.80
**II**	9750	−54.86	1.64	0.38
**III**	4300	−45.50	1.46	0.14
**IV**	800	−41.71	1.81	0
**V**	100	−35.51	2.94	0
**VI**	1750	−53.98	3.03	0.59
**VII**	1400	−63.28	7.12	0.43

**Table 4:** Parameters for the different considered wireless communication systems.

		Transmitted Power	Sensitivity
**ZigBee XBee Pro**		10 dBm	−100 dBm
**ZigBee XBee**		10 dBm	−92 dBm
**BLE**	**Minimum Transmitted Power**	−20 dBm	−93 dBm
	**Maximum Transmitted Power**	10 dBm	−87 dBm
**Bluetooth Class 1**		20 dBm	−90 dBm
**Bluetooth Class 2**		4 dBm	−90 dBm
**Bluetooth Class 3**		0 dBm	−90 dBm
**802.11p**	**BPSK–3 Mbps**	24 dBm	−95 dBm
	**16 QAM–18 Mbps**	24 dBm	−83 dBm
	**64 QAM–27 Mbps**	21 dBm	−77 dBm

**Table 5:** Congestion level service rules.

		Traversing Vehicles Rate		
		High	Medium	Low
#waitingGreen	High	CTh = 5	CTh = 7	CTh = 10
	Medium	CTh = 3	CTh = 5	CTh = 7
	Low	CTh = 1	CTh = 2	CTh = 3

## Abbreviations

The following abbreviations are used in this manuscript:

WSN	Wireless Sensor Network
ITS	Intelligent Transportation Systems
ISM	Industrial, Scientific and Medical
V2V	Vehicle to Vehicle
V2I	Vehicle to Infrastructure
ICA	Intersection Control Agent
VANET	Vehicular Ad-Hoc Network
RFID	Radio Frequency Identification
RL	Ray Launching
RT	Ray Tracing
MoM	Method of Moments
FDTD	Finite Difference Time Domain
GO	Geometrical Optics
GTD	Geometrical Theory of Diffraction
UTD	Uniform Theory of Diffraction
BER	Bit Error Rate
BLE	Bluetooth Low Energy
WAVE	Wireless Access in Vehicular Environments
AR	Arrival Rate
PR	Processing Rate
CTh	Congestion Threshold
